# Targeting innate and adaptive immunity to suppress lung cancer metastasis

**DOI:** 10.3389/fimmu.2025.1662754

**Published:** 2025-11-27

**Authors:** Rong Qin

**Affiliations:** Department of Pathology, Xiangyang No.1 People’s Hospital, Hubei University of Medicine, Xiangyang, China

**Keywords:** lung cancer, adaptive immune cells, innate immune cells, immune dysregulation, metastasis, therapeutic target

## Abstract

Lung cancer remains the leading cause of cancer-related mortality globally, with metastasis and recurrence as the primary determinants of poor prognosis. Despite advances in immunotherapy, intrinsic and acquired resistance to immune checkpoint inhibitors (ICIs) underscores the need to explore alternative immunomodulatory strategies. Emerging evidence highlights the critical yet dual roles of innate and adaptive immune cells within the tumor microenvironment (TME) in either restraining or facilitating metastatic dissemination. Adaptive immunity, dominated by T and B cells, orchestrates context-dependent antitumor responses or immunosuppression, while innate immune dysregulation fosters metastatic niches. We highlight translational opportunities, such as natural killer (NK) cell activation, macrophage reprogramming, and dendritic cell (DC)-based vaccines, alongside prognostic biomarkers like peripheral NK activity and tryptase^+^ mast cell infiltration. This review summarizes the interplay of immune cell subsets, including T and B lymphocytes, macrophages, DCs, NK cells, and mast cells, in lung cancer progression. By synthesizing preclinical and clinical insights, this review identifies unresolved challenges and proposes targeting innate immunity as a promising avenue to augment current therapies and mitigate metastasis.

## 1 Introduction

Primary lung cancer remains one of the most prevalent and lethal malignancies worldwide (1, 2). Although recent data indicate a decline in its proportional contribution to overall cancer mortality attributed to advances in surgery, radiotherapy, chemotherapy, targeted therapies, and immunotherapy (3, 4), lung cancer continues to lead in both incidence and mortality. Metastasis and recurrence remain the dominant causes of poor outcomes, yet widely accepted theoretical frameworks and effective strategies for preventing dissemination are still lacking. The advent of immune checkpoint inhibitors (ICIs) has transformed clinical care, offering durable responses in some patients. However, many individuals exhibit primary resistance or develop immune escape over time, limiting therapeutic efficacy (5).

Emerging evidence suggests that adaptive immune cells predominate within the lung cancer microenvironment, while innate immune components such as natural killer cells, macrophages, granulocytes, monocytes, dendritic cells, and mast cells are significantly underrepresented compared to non-tumor lung tissue. This relative depletion implies a role for innate immune dysfunction in facilitating metastasis (6). This review systematically summarizes the roles of diverse immune subsets in lung cancer invasion and dissemination, aiming to uncover novel immunological targets for therapeutic development.

## 2 Adaptive immune cells in the lung cancer microenvironment

### 2.1 T lymphocyte subsets

Patients with cancer exhibit impaired immune surveillance, enabling tumor immune evasion through diverse mechanisms (7). In lung cancer, T lymphocytes dominate the tumor microenvironment (TME), where CD4^+^ T cells coordinate immune responses via cytokine secretion, while CD8^+^ cytotoxic T lymphocytes (CTLs) eliminate neoplastic cells expressing tumor neoantigens (8). In the pulmonary TME, dysregulated immune profiles—marked by decreased CD4^+^, increased CD8^+^, and a reduced CD4^+^/CD8^+^ ratio—are observed in lung cancer patients (9, 10). Regulatory T cells (Tregs), a CD4^+^ subset defined by CD4^+^/CD25^high^/FoxP3^+^/CD127^−^ markers, play a key immunosuppressive role by inhibiting T, dendritic, and natural killer (NK) cell functions (11, 12). Tregs are significantly enriched in the BALF of lung cancer patients—particularly those with non-squamous subtypes such as adenocarcinoma—compared to benign conditions (13). Clinically, an elevated Treg/CD8^+^ ratio has been consistently associated with poorer overall survival and reduced responses to ICIs (14, 15), underscoring the prognostic value of T-cell profiling in both early- and late-stage lung cancer (16). Moreover, variations in T-cell infiltration and exhaustion signatures across ethnic and histological subgroups suggest that population diversity may influence immune responsiveness and therapeutic benefit. Thus, Treg quantification may reflect local immunosuppression, informing prognosis and immunotherapy stratification (17). Beyond diagnostics, T cells offer therapeutic potential. Xiao et al. (18) reviewed four generations of chimeric antigen receptor T (CAR-T) cell therapy in lung cancer, highlighting key targets including EGFR, EphA2, MUC1, and HER2, along with associated toxicities. These insights underscore the diagnostic, prognostic, and therapeutic utility of T lymphocytes in lung cancer immuno-oncology.

### 2.2 B lymphocytes

Tumor-infiltrating B lymphocytes (TIL-Bs) represent a crucial adaptive immune component in lung cancer, exhibiting dual, context-dependent functions in both tumor suppression and promotion (19). Circulating B cells secrete cytokines and differentiate into Be1/Be2 subsets, paralleling Th1/Th2 profiles and shaping immune polarization (20–22). In lung squamous carcinoma, tumor-associated antigens such as SCCA can drive B-cell-mediated antibody production and formation of circulating immune complexes (CICs), which activate FcγR signaling in myeloid cells, thereby recruiting leukocytes into the TME and facilitating progression and metastasis (23). Pharmacological inhibition of B-cell activation or interference with B-cell-driven innate responses may thus curb malignant transformation of precancerous lesions. Conversely, TIL-Bs can elicit potent antitumor responses through enhancing CD4^+^ memory T-cell formation, supporting cytotoxic T-cell function, and orchestrating TLS, which are associated with improved prognosis and immune activation. Local delivery of cytokines such as CXCL13 or lymphotoxin enhances TLS formation and TIL-B recruitment, strengthening vaccine responses (24). In certain NSCLC subtypes, TIL-Bs may differentiate into IgG4-secreting plasma cells contributing to tumor control (25). They also generate tumor-specific antibodies forming *in situ* immune complexes with direct cytotoxicity. Intratumoral germinal centers identified by Michael et al. (26) suggest B-cell-driven local immunity, with memory B-cell-derived antibody cloning offering therapeutic potential (27). Furthermore, some evidence suggests that TIL-Bs possess direct cytotoxic capacity against tumor cells via the TRAIL/Apo1 signaling pathway (28). High TLS density correlates with improved survival, notably in female patients and adenocarcinoma subtypes, underscoring sex- and histology-dependent differences in B-cell-mediated immunity (29, 30).

## 3 Innate immune cells in the lung cancer microenvironment

Innate immune dysregulation is pivotal in lung cancer recurrence and metastasis. Clinical studies link peripheral monocyte and neutrophil counts, as well as cytotoxic receptor transcripts, to overall survival (31). Notably, NK-cell dynamics illustrate this complexity: in early-stage disease, expansion of peripheral cytotoxic CD56^dim^ NK subsets expressing CD16 and NCRs signals active immune surveillance (32, 33). However, as the disease advances, NK cells fail to maintain immune clearance and homeostasis, resulting in immune escape and subsequent metastasis (34). Importantly, reduced intratumoral NK-cell density has been linked with shorter disease-free survival and diminished response to immunotherapy, whereas higher baseline NK activity in peripheral blood correlates with improved outcomes, highlighting their translational value as predictive biomarkers across diverse patient populations (35, 36). High density of CD68^+^ macrophages/monocytes has a positive correlation with reduced mortality in lung cancer patients. Beyond the primary tumor, innate immune cells are critical in establishing pre-metastatic niches. Primary tumor cells reprogram distant organs by recruiting myeloid progenitor cells and modulating the secretion of cytokines, soluble factors, and extracellular vesicles, thereby fostering a permissive microenvironment enriched with neutrophils and alveolar macrophages conducive to metastatic colonization (37).

### 3.1 Macrophages in the lung cancer microenvironment

Macrophages, key constituents of the innate immune system, are broadly categorized into classically activated (M1) and alternatively activated (M2) phenotypes (38). M1 macrophages, induced by IFN-γ, TNF, or LPS, secrete high levels of TNF, IL-12, and IL-23, driving Th1-mediated inflammation and exerting antitumor effects. In contrast, M2 macrophages, stimulated by IL-4 or IL-13, release IL-10 and various chemokines that promote Th2 responses, tissue remodeling, angiogenesis, and immunosuppression (39). In lung cancer, tumor-associated macrophages (TAMs) are predominantly M2-like, supporting tumor progression. Studies suggest that M2-polarized TAMs enhance tumor invasion and metastasis by upregulating VEGF-C and its receptor VEGFR3, thereby driving angiogenesis and lymphangiogenesis (40). Additionally, M2-polarized TAMs secrete matrix-remodeling enzymes such as MMP-2, which facilitate tumor dissemination (41), while their production of IL-10 suppresses pro-inflammatory cytokines (TNF-α, IL-12, IL-1) and promotes tumor immune escape (42, 43). Quantification and classification of TAMs, particularly CD163^+^ M2 macrophages, are useful prognostic indicators in NSCLC. Elevated CD163^+^ cell counts are associated with disease progression. Moreover, NSCLC cells may recruit M2-like TAMs via VEGF, and this axis can be interrupted using anti-VEGF monoclonal antibodies (bevacizumab) (44). Therapeutic interventions against M2-polarized TAMs encompass the inhibition of chemokines, including CCL2, CCL7, and CCL8, to limit their recruitment, suppression of M2 polarization pathways, and reprogramming of M2-like TAMs into pro-inflammatory M1-like phenotypes (45–47).

### 3.2 Mast cells

Mast cells, another integral component of innate immunity, contribute to tumor growth and metastasis across multiple cancer types. In lung cancer, mast cells promote tumor progression via pro-angiogenic signaling, autocrine hormone production, and release of growth factors (48–51). Degranulation products facilitate cervical cancer metastasis (52), and histamine release has been linked to colorectal cancer severity (53). In NSCLC, intratumoral mast cells are prevalent within tumor stroma and correlate with patient survival (54). Human mast cells exhibit two phenotypes: MCT (tryptase-positive) and MCTC (tryptase- and chymase-positive). MCT predominates in mucosal tissues, while MCTC localizes to dermal and connective tissues. Both subtypes are associated with improved NSCLC prognosis, suggesting a potential antitumor role. Mast cells may enhance antitumor immunity by secreting TNF-α, which promotes T-cell proliferation, and in turn, TNF-α-stimulated T cells support mast cell expansion through a positive feedback loop (55). Furthermore, proteases released during degranulation disrupt the tumor extracellular matrix, thereby restraining tumor growth (54).

### 3.3 Dendritic cells

Dendritic cells (DCs) are the most potent professional antigen-presenting cells, capable of antigen uptake, processing, and presentation to initiate adaptive immunity. Immature DCs exhibit strong migratory capabilities, while mature DCs efficiently prime naive T cells, orchestrating immune responses. DCs are found in epithelial tissues interfacing with the environment, including the skin, nasal mucosa, lungs, and gastrointestinal tract, and in circulation as precursors. Activated DCs migrate to lymphoid organs to interact with T and B cells. Genetically modified DCs expressing CCL21 can recruit naive T cells and promote their differentiation into tumor-specific cytotoxic lymphocytes (56). In addition, DCs secrete chemokines such as CCL1 and CCL17 to enhance CD8^+^ T-cell activation (57). Plasmacytoid dendritic cells (pDCs), a distinct subset, bridge innate and adaptive immunity through antigen presentation and modulation of NK-, T-, and B-cell activity. pDCs can either induce immune tolerance or stimulate immunity depending on cytokine signals. Stimulation of pDCs with CTLA4-Ig or OX2 (CD200) induces indoleamine 2,3-dioxygenase expression, suppressing T-cell proliferation and promoting tolerance (58). Many tumors harbor abundant immature DCs and pDCs, which contribute to tumor metastasis and recurrence. In breast cancer, pDC-expressed ICOS ligand facilitates CD4^+^ T-cell-mediated immunosuppression and tumor growth (59). Conversely, TLR agonists can trigger pDCs to secrete type I interferons, activate intratumoral immature DCs, and initiate anti-angiogenic, tumor-specific T-cell responses. Imiquimod-stimulated pDCs have demonstrated efficacy in melanoma by enhancing T-cell-mediated immunity (60). However, research on pDCs in lung cancer remains limited and warrants further investigation.

### 3.4 Natural killer cells

**Figure 1:**
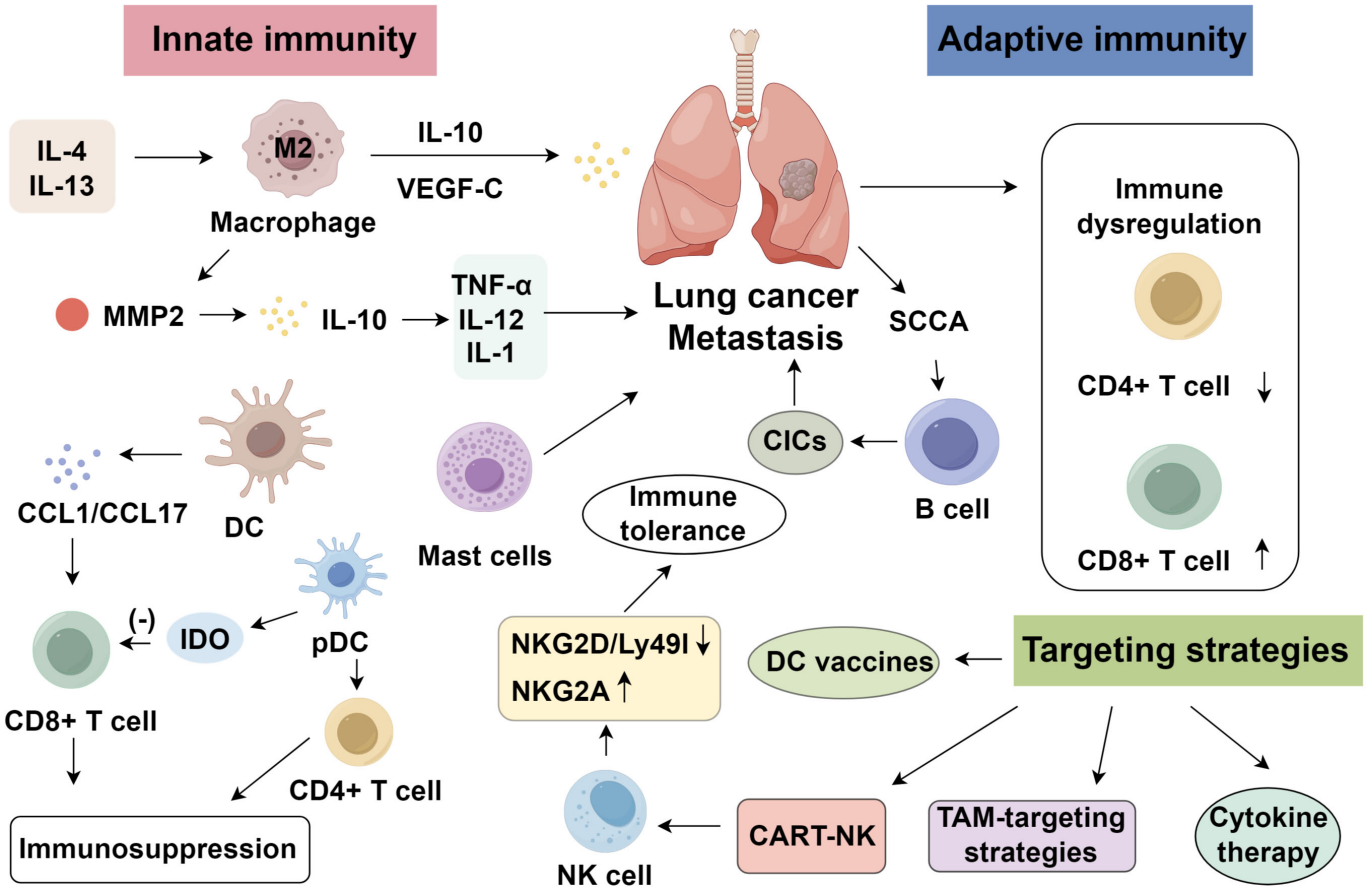
Roles of innate and adaptive immunity in lung cancer.

NK cells are large granular lymphocytes that mediate cytotoxicity against tumor and pathogen-infected cells without prior sensitization and independently of MHC restriction. In lung cancer, the extent of NK-cell infiltration correlates with tumor subtype, smoking history, tumor size, and prognosis (61). Greater NK-cell presence is observed in squamous cell carcinoma relative to adenocarcinoma (62). Non-smokers show higher NK infiltration than smokers, and tumors with greater NK density exhibit improved clinical outcomes (63). In the lung tumor microenvironment, inhibitory NK receptors are upregulated, while activating receptors are downregulated. Murine models suggest that downregulation of stimulatory receptors NKG2D and Ly49I, along with upregulation of inhibitory NKG2A, may underlie tumor-induced immune tolerance. NKG2D enhances antitumor immunity through perforin-mediated apoptosis, while Ly49I aids NK-mediated cytolysis. In NSCLC patients, combined chemotherapy and NK-cell reinfusion prolonged median survival compared to chemotherapy alone (64). Tumoral expression of NKG2D ligand MICA may predict response to NK-based immunotherapy. Moreover, gefitinib has been shown to enhance NK cytotoxicity against lung cancer cells (65). Previous IL-2-based NK therapies were limited by toxicity and Treg expansion (66). Recent advances have enabled NK-cell engineering with tumor-specific chimeric antigen receptors, yielding promising antitumor effects (67). Additionally, bispecific proteins targeting both tumor antigens and NK-activating receptors have demonstrated targeted cytotoxicity through antibody-dependent cellular cytotoxicity (ADCC) (68) (**Figure 1**).

### 3.5 Neutrophils

Neutrophils are among the earliest responders in the tumor microenvironment and have emerged as critical regulators of lung cancer metastasis as well as metastatic colonization within the lung (69). Tumor-associated neutrophils (TANs) display functional plasticity, broadly polarized into antitumor (N1) and protumor (N2) phenotypes (70, 71). While N1 neutrophils exert cytotoxic activity through ROS, TNF-α, and direct tumor cell killing, N2 neutrophils promote angiogenesis, extracellular matrix remodeling, and immunosuppression, thereby facilitating metastatic dissemination (72–75). A particularly important mechanism is the formation of neutrophil extracellular traps (NETs), which create a fibrous scaffold that captures CTCs, enhancing their adhesion and colonization in distant sites such as the lung (76–78). Preclinical studies have shown that tumor-derived factors, including G-CSF, IL-8, and TGF-β, drive neutrophil mobilization and N2 polarization, supporting metastatic niche formation (70, 79, 80). Clinically, an elevated neutrophil-to-lymphocyte ratio has been consistently associated with poor outcomes in NSCLC, reinforcing its prognostic value (81–83). Therapeutic strategies targeting neutrophils include inhibition of CXCR1/2 to block IL-8-mediated recruitment, disruption of NETs with DNase or PAD4 inhibitors, and reprogramming TANs toward antitumor phenotypes with TGF-β blockade (84, 85). These approaches, in combination with ICIs or NK-based therapies, may synergistically suppress metastatic spread by dismantling neutrophil-driven pre-metastatic niches (86, 87). Collectively, neutrophils represent both biomarkers of disease progression and actionable targets within innate immune-based therapeutic strategies.

## 4 Clinical applications of innate immune cells in lung cancer

### 4.1 Targeting innate immune cells to suppress lung cancer metastasis

NK-cell-based immunotherapies are central to modulating metastasis, with significant progress in hematologic malignancies, though efficacy in solid tumors like lung cancer remains under investigation (88). Among cytokines, IL-15 and its receptor agonists show high clinical safety, while monoclonal antibodies targeting NK inhibitory receptors—anti-KIR (IPH2101, lirilumab) and anti-NKG2A (monalizumab)—are in clinical trials (89). Adoptive NK therapies, including NK-92 and CAR-NK cells, demonstrate efficacy in suppressing lung cancer metastasis. Similarly, γδ T- and iNKT-cell therapies exhibit antitumor effects in animal models, though clinical benefits are limited (90). TAMs suppress CD8^+^ T-cell cytotoxicity via physical interactions, making TAM-targeting strategies—through depletion, reprogramming, or blockade of functional molecules—attractive for metastasis control (91). Antibodies against CCR2, CSF1R, and IL-1β reduce TAM recruitment, survival, and polarization, improving the immunosuppressive tumor microenvironment (92). DCs, as key APCs, are being leveraged to enhance antigen presentation and elicit robust T-cell responses. Personalized DC vaccines transfected with tumor-associated antigen (TAA) mRNAs have shown favorable survival benefits in advanced lung cancer patients without adverse effects (93).

In addition to these approaches, neoantigen-based vaccines and adoptive cell transfer (ACT) therapies have emerged as critical components of next-generation immunotherapy in lung cancer (94–96). Neoantigen vaccines, derived from tumor-specific mutations, can induce highly personalized T-cell responses with minimal risk of autoimmunity (97). Early-phase clinical trials have demonstrated that neoantigen-pulsed dendritic cells or peptide vaccines can elicit durable antitumor immunity in subsets of patients with NSCLC (97, 98). Likewise, ACT therapies, including TILs, CAR-T cells, and CAR-NK cells, have shown encouraging activity in targeting lung cancer-associated antigens such as EGFR, MUC1, and mesothelin (99–102). Although challenges such as antigen heterogeneity, limited trafficking into solid tumors, and immune-related toxicities remain, combinatorial strategies integrating ACT with innate immune modulation (NK activation or TAM reprogramming) represent a promising avenue to overcome resistance and suppress metastasis (103–105).

### 4.2 Innate immune cells as prognostic biomarkers in lung cancer

Monitoring innate immune cell populations and related mediators in peripheral blood provides novel avenues for prognostic assessment in lung cancer (106). For example, baseline NK-cell activity in peripheral blood prior to immunotherapy has demonstrated predictive value for therapeutic response in NSCLC, with higher NK-cell activity positively correlating with progression-free survival. This metric exhibits a sensitivity of 80% and a specificity of 68.4%, making it a robust predictive tool for immunotherapeutic outcomes (35). Importantly, the strength of this association appears highly context-dependent: enhanced NK-cell activity correlates more consistently with clinical benefit in tumors exhibiting immunogenic characteristics, such as elevated PD-L1 expression or high tumor mutational burden, and in histological subtypes characterized by greater NK infiltration, which is more frequently observed in squamous carcinoma than in adenocarcinoma (107, 108). Radiotherapeutic efficacy in lung cancer is also influenced by circulating neutrophil counts. Although pre-radiotherapy neutrophil levels are not directly associated with prognosis, elevated neutrophil populations can contribute to radiotherapy resistance (109). In clinical practice, neutrophil-forward composite indices (neutrophil-to-lymphocyte ratio) often co-vary with M2-like macrophage signatures and systemic inflammation; together, these profiles align with inferior PFS/OS and reduced ICI responsiveness, reinforcing their utility as low-cost risk stratifiers that complement tumor-intrinsic markers (110, 111). Furthermore, infiltration of tryptase^+^ MCs has been proposed as a potential prognostic biomarker for lung cancer metastasis. High intratumoral densities of tryptase^+^ MCs correlate significantly with lymph node metastasis and are associated with both overall and progression-free survival (112). These findings collectively highlight the emerging utility of innate immune cells as biomarkers for lung cancer metastasis. However, further mechanistic and clinical investigations are warranted to validate their prognostic value.

## 5 Limitations and challenges

Despite the rapid advances in antigen presentation immunotherapy for lung cancer, several limitations constrain its clinical efficacy. First, ICIs are effective only in a subset of patients, with intrinsic and acquired resistance remaining prevalent (113, 114). Mechanisms underlying resistance include impaired upregulation of alternative inhibitory pathways and recruitment of immunosuppressive cells such as Tregs and myeloid-derived suppressor cells (115, 116). Second, patient heterogeneity—arising from diverse genetic, epigenetic, and immunological profiles—limits the predictive accuracy of current biomarkers such as PD-L1 expression and tumor mutational burden (117, 118). This variability complicates patient stratification and response prediction. Third, immunotherapy is often accompanied by immune-related adverse events, including pneumonitis, colitis, and endocrinopathies, which can significantly affect patient quality of life and limit treatment continuity (119, 120). Overcoming these challenges will require rational combination strategies (ICIs with TAM or NK-targeted therapies), integration of precision biomarkers, and the development of novel immunomodulatory agents that minimize toxicity while enhancing efficacy (102, 121). Addressing these barriers is critical for optimizing the therapeutic potential of immune-based interventions in metastatic lung cancer.

## 6 Conclusion

The immune landscape of lung cancer is a dynamic interplay of pro- and anti-metastatic signals mediated by diverse immune cell populations. While adaptive immunity, particularly cytotoxic T cells and tertiary lymphoid structure-associated B cells, forms the backbone of antitumor responses, innate immune cells are increasingly recognized as pivotal regulators of metastatic dissemination. Key findings reveal that NK-cell dysfunction, M2 macrophage polarization, and tolerogenic DC activity contribute to immune evasion, whereas reinvigorating innate cytotoxicity or reprogramming immunosuppressive niches may offer therapeutic leverage. Clinically, innate immune biomarkers such as peripheral NK-cell activity and mast cell density exhibit prognostic potential, though their mechanistic underpinnings require further validation.

Future investigations should prioritize integrated preclinical and clinical approaches to validate these strategies. At the experimental level, murine lung cancer models and patient-derived xenografts could be used to test macrophage-reprogramming interventions or NK-cell engineering platforms. Multi-omics technologies, including single-cell and spatial transcriptomics, may further dissect the crosstalk between innate and adaptive compartments during metastasis. At the clinical level, early-phase trials combining CAR-NK therapy or DC-based vaccines with immune checkpoint inhibitors should be explored to assess synergistic efficacy. Additionally, longitudinal biomarker studies measuring peripheral NK activity, TAM phenotypes, and mast cell infiltration could refine patient stratification and therapeutic monitoring. Addressing these gaps will be essential to translate innate immune insights into effective therapies for metastatic lung cancer.

## References

[1] Zhou J Wan F Xiao B Li X Peng C Peng FU . Metochalcone induces senescence-associated secretory phenotype via JAK2/STAT3 pathway in breast cancer. Oncol Res. (2024) 32:943–53. doi: 10.32604/or.2023.044775, PMID: 38686052 PMC11055985

[2] Huang X Chen C Chen Y Zhou H Chen Y Huang Z . Silencing of the long non-coding RNA LINC00265 triggers autophagy and apoptosis in lung cancer by reducing protein stability of SIN3A oncogene. Oncol Res. (2024) 32:1185–95. doi: 10.32604/or.2023.030771, PMID: 38948024 PMC11211643

[3] Zhang J Tan Y Shou Y Fang L Li X Lai J . The relationship between adjuvant radiotherapy and survival of osteosarcoma: a case-control study. Biotechnol Genet Eng Rev. (2023) 39:796–809. doi: 10.1080/02648725.2022.2163805, PMID: 36596221

[4] Araghi M Mannani R Heidarnejad Maleki A Hamidi A Rostami S Safa SH . Recent advances in non-small cell lung cancer targeted therapy; an update review. Cancer Cell Int. (2023) 23:162. doi: 10.1186/s12935-023-02990-y, PMID: 37568193 PMC10416536

[5] Mariniello A Borgeaud M Weiner M Frisone D Kim F Addeo A . Primary and acquired resistance to immunotherapy with checkpoint inhibitors in NSCLC: from bedside to bench and back. BioDrugs. (2025) 39:215–35. doi: 10.1007/s40259-024-00700-2, PMID: 39954220 PMC11906525

[6] Stankovic B Bjørhovde HAK Skarshaug R Aamodt H Frafjord A Müller E . Immune cell composition in human non-small cell lung cancer. Front Immunol. (2018) 9:3101. doi: 10.3389/fimmu.2018.03101, PMID: 30774636 PMC6367276

[7] Sun LU Tan H Yu T Liang R . Identification of lncRNAs associated with T cells as potential biomarkers and therapeutic targets in lung adenocarcinoma. Oncol Res. (2023) 31:967–88. doi: 10.32604/or.2023.042309, PMID: 37744265 PMC10513944

[8] Wu Y Yuan M Wang C Chen Y Zhang Y Zhang J . T lymphocyte cell: A pivotal player in lung cancer. Front Immunol. (2023) 14:1102778. doi: 10.3389/fimmu.2023.1102778, PMID: 36776832 PMC9911803

[9] Wang WJ Tao Z Gu W Sun LH . Variation of blood T lymphocyte subgroups in patients with non- small cell lung cancer. Asian Pac J Cancer Prev. (2013) 14:4671–3. doi: 10.7314/APJCP.2013.14.8.4671, PMID: 24083723

[10] Xie H Xi X Lei T Liu H Xia Z . CD8(+) T cell exhaustion in the tumor microenvironment of breast cancer. Front Immunol. (2024) 15:1507283. doi: 10.3389/fimmu.2024.1507283, PMID: 39717767 PMC11663851

[11] Nedoszytko B Lange M Sokołowska-Wojdyło M Renke J Trzonkowski P Sobjanek M . The role of regulatory T cells and genes involved in their differentiation in pathogenesis of selected inflammatory and neoplastic skin diseases. Part I: Treg properties and functions. Postepy Dermatol Alergol. (2017) 34:285–94. doi: 10.5114/ada.2017.69305, PMID: 28951701 PMC5560174

[12] Ge J Yin X Chen L . Regulatory T cells: masterminds of immune equilibrium and future therapeutic innovations. Front Immunol. (2024) 15:1457189. doi: 10.3389/fimmu.2024.1457189, PMID: 39290699 PMC11405253

[13] Mariniello A Tabbò F Indellicati D Tesauro M Rezmives NA Reale ML . Comparing T cell subsets in Broncho-Alveolar lavage (BAL) and peripheral blood in patients with advanced lung cancer. Cells. (2022) 11:3226. doi: 10.3390/cells11203226, PMID: 36291098 PMC9600421

[14] Denize T Jegede OA Matar S El Ahmar N West DJ Walton E . PD-1 expression on intratumoral regulatory T cells is associated with lack of benefit from anti-PD-1 therapy in metastatic clear-cell renal cell carcinoma patients. Clin Cancer Res. (2024) 30:803–13. doi: 10.1158/1078-0432.CCR-23-2274, PMID: 38060202 PMC10922154

[15] Alonso-Miguel D Valdivia G Guerrera D Perez-Alenza MD Pantelyushin S Alonso-Diez A . Neoadjuvant in situ vaccination with cowpea mosaic virus as a novel therapy against canine inflammatory mammary cancer. J Immunother Cancer. (2022) 10:e004044. doi: 10.1136/jitc-2021-004044, PMID: 35277459 PMC8919457

[16] Hu X Estecio MR Chen R Reuben A Wang L Fujimoto J . Evolution of DNA methylome from precancerous lesions to invasive lung adenocarcinomas. Nat Commun. (2021) 12:687. doi: 10.1038/s41467-021-20907-z, PMID: 33514726 PMC7846738

[17] Khambholja K Gehani M Kothari R Marulkar S . Prognostic value of tumour-associated regulatory T-cells as a biomarker in non-small cell lung cancer: a systematic review and meta-analysis. Syst Rev. (2024) 13:233. doi: 10.1186/s13643-024-02642-w, PMID: 39272135 PMC11401299

[18] Xiao BF Zhang JT Zhu YG Cui XR Lu ZM Yu BT . Chimeric antigen receptor T-cell therapy in lung cancer: potential and challenges. Front Immunol. (2021) 12:782775. doi: 10.3389/fimmu.2021.782775, PMID: 34790207 PMC8591168

[19] Yang H Zhang Z Li J Wang K Zhu W Zeng Y . The dual role of B cells in the tumor microenvironment: implications for cancer immunology and therapy. Int J Mol Sci. (2024) 25:11825. doi: 10.3390/ijms252111825, PMID: 39519376 PMC11546796

[20] de Visser KE Korets LV Coussens LM . *De novo* carcinogenesis promoted by chronic inflammation is B lymphocyte dependent. Cancer Cell. (2005) 7:411–23. doi: 10.1016/j.ccr.2005.04.014, PMID: 15894262

[21] Harris DP Haynes L Sayles PC Duso DK Eaton SM Lepak NM . Reciprocal regulation of polarized cytokine production by effector B and T cells. Nat Immunol. (2000) 1:475–82. doi: 10.1038/82717, PMID: 11101868

[22] Gabrilovich DI Ostrand-Rosenberg S Bronte V . Coordinated regulation of myeloid cells by tumours. Nat Rev Immunol. (2012) 12:253–68. doi: 10.1038/nri3175, PMID: 22437938 PMC3587148

[23] Andreu P Johansson M Affara NI Pucci F Tan T Junankar S . FcRgamma activation regulates inflammation-associated squamous carcinogenesis. Cancer Cell. (2010) 17:121–34. doi: 10.1016/j.ccr.2009.12.019, PMID: 20138013 PMC3082507

[24] Siliņa K Rulle U Kalniņa Z Linē A . Manipulation of tumour-infiltrating B cells and tertiary lymphoid structures: a novel anti-cancer treatment avenue? Cancer Immunol Immunother. (2014) 63:643–62. doi: 10.1007/s00262-014-1544-9, PMID: 24695950 PMC11029173

[25] Fujimoto M Yoshizawa A Sumiyoshi S Sonobe M Kobayashi M Koyanagi I . Stromal plasma cells expressing immunoglobulin G4 subclass in non-small cell lung cancer. Hum Pathol. (2013) 44:1569–76. doi: 10.1016/j.humpath.2013.01.002, PMID: 23465276

[26] Campa MJ Moody MA Zhang R Liao HX Gottlin EB Patz EF Jr . Interrogation of individual intratumoral B lymphocytes from lung cancer patients for molecular target discovery. Cancer Immunol Immunother. (2016) 65:171–80. doi: 10.1007/s00262-015-1787-0, PMID: 26739486 PMC11028467

[27] Yasuda M Takenoyama M Obata Y Sugaya M So T Hanagiri T . Tumor-infiltrating B lymphocytes as a potential source of identifying tumor antigen in human lung cancer. Cancer Res. (2002) 62:1751–6., PMID: 11912150

[28] Kemp TJ Moore JM Griffith TS . Human B cells express functional TRAIL/Apo-2 ligand after CpG-containing oligodeoxynucleotide stimulation. J Immunol. (2004) 173:892–9. doi: 10.4049/jimmunol.173.2.892, PMID: 15240676

[29] Sun X Liu W Sun L Mo H Feng Y Wu X . Maturation and abundance of tertiary lymphoid structures are associated with the efficacy of neoadjuvant chemoimmunotherapy in resectable non-small cell lung cancer. J Immunother Cancer. (2022) 10:e005531. doi: 10.1136/jitc-2022-005531, PMID: 37011953 PMC9644367

[30] Germain C Devi-Marulkar P Knockaert S Biton J Kaplon H Letaïef L . Tertiary lymphoid structure-B cells narrow regulatory T cells impact in lung cancer patients. Front Immunol. (2021) 12:626776. doi: 10.3389/fimmu.2021.626776, PMID: 33763071 PMC7983944

[31] Riemann D Turzer S Ganchev G Schütte W Seliger B Möller M . Monitoring blood immune cells in patients with advanced small cell lung cancer undergoing a combined immune checkpoint inhibitor/chemotherapy. Biomolecules. (2023) 13:190. doi: 10.3390/biom13020190, PMID: 36830562 PMC9953684

[32] Charrier M Mezquita L Lueza B Dupraz L Planchard D Remon J . Circulating innate immune markers and outcomes in treatment-naïve advanced non-small cell lung cancer patients. Eur J Cancer. (2019) 108:88–96. doi: 10.1016/j.ejca.2018.12.017, PMID: 30648633

[33] Wessel RE Ageeb N Obeid JM Mauldin IS Goundry KA Hanson GF . Spatial colocalization and combined survival benefit of natural killer and CD8 T cells despite profound MHC class I loss in non-small cell lung cancer. J Immunother Cancer. (2024) 12:e009126. doi: 10.1136/jitc-2024-009126, PMID: 39299754 PMC11418484

[34] Sun H Sun C . The rise of NK cell checkpoints as promising therapeutic targets in cancer immunotherapy. Front Immunol. (2019) 10:2354. doi: 10.3389/fimmu.2019.02354, PMID: 31681269 PMC6812684

[35] Choi MG Kim YJ Lee JC Rho JK Choi CM . Efficacy of natural killer cell activity as a biomarker for predicting immunotherapy response in non-small cell lung cancer. Thorac Cancer. (2020) 11:3337–45. doi: 10.1111/1759-7714.13677, PMID: 33017518 PMC7606014

[36] Cho YH Choi MG Kim DH Choi YJ Kim SY Sung KJ . Natural killer cells as a potential biomarker for predicting immunotherapy efficacy in patients with non-small cell lung cancer. Target Oncol. (2020) 15:241–7. doi: 10.1007/s11523-020-00712-2, PMID: 32285316

[37] Milette S Fiset PO Walsh LA Spicer JD Quail DF . The innate immune architecture of lung tumors and its implication in disease progression. J Pathol. (2019) 247:589–605. doi: 10.1002/path.5241, PMID: 30680732

[38] Zhai X Zhang H Xia Z Liu M Du G Jiang Z . Oxytocin alleviates liver fibrosis via hepatic macrophages. JHEP Rep. (2024) 6:101032. doi: 10.1016/j.jhepr.2024.101032, PMID: 38882603 PMC11177191

[39] Ngambenjawong C Gustafson HH Pun SH . Progress in tumor-associated macrophage (TAM)-targeted therapeutics. Adv Drug Delivery Rev. (2017) 114:206–21. doi: 10.1016/j.addr.2017.04.010, PMID: 28449873 PMC5581987

[40] Hwang I Kim JW Ylaya K Chung EJ Kitano H Perry C . Tumor-associated macrophage, angiogenesis and lymphangiogenesis markers predict prognosis of non-small cell lung cancer patients. J Transl Med. (2020) 18:443. doi: 10.1186/s12967-020-02618-z, PMID: 33228719 PMC7686699

[41] Zhu R Huang J Qian F . The role of tumor-associated macrophages in lung cancer. Front Immunol. (2025) 16:1556209. doi: 10.3389/fimmu.2025.1556209, PMID: 40079009 PMC11897577

[42] Astekar M Metgud R Sharma A Soni A . Hidden keys in stroma: Unlocking the tumor progression. J Oral Maxillofac Pathol. (2013) 17:82–8. doi: 10.4103/0973-029X.110742, PMID: 23798836 PMC3687195

[43] Rhee I . Diverse macrophages polarization in tumor microenvironment. Arch Pharm Res. (2016) 39:1588–96. doi: 10.1007/s12272-016-0820-y, PMID: 27562774

[44] Ohri CM Shikotra A Green RH Waller DA Bradding P . The tissue microlocalisation and cellular expression of CD163, VEGF, HLA-DR, iNOS, and MRP 8/14 is correlated to clinical outcome in NSCLC. PLoS One. (2011) 6:e21874. doi: 10.1371/journal.pone.0021874, PMID: 21799753 PMC3142113

[45] Tyagi A Singh RP Ramasamy K Raina K Redente EF Dwyer-Nield LD . Growth inhibition and regression of lung tumors by silibinin: modulation of angiogenesis by macrophage-associated cytokines and nuclear factor-kappaB and signal transducers and activators of transcription 3. Cancer Prev Res (Phila). (2009) 2:74–83. doi: 10.1158/1940-6207.CAPR-08-0095, PMID: 19139021 PMC2615181

[46] Duluc D Delneste Y Tan F Moles MP Grimaud L Lenoir J . Tumor-associated leukemia inhibitory factor and IL-6 skew monocyte differentiation into tumor-associated macrophage-like cells. Blood. (2007) 110:4319–30. doi: 10.1182/blood-2007-02-072587, PMID: 17848619

[47] Nakanishi Y Nakatsuji M Seno H Ishizu S Akitake-Kawano R Kanda K . COX-2 inhibition alters the phenotype of tumor-associated macrophages from M2 to M1 in ApcMin/+ mouse polyps. Carcinogenesis. (2011) 32:1333–9. doi: 10.1093/carcin/bgr128, PMID: 21730361

[48] Takanami I Takeuchi K Naruke M . Mast cell density is associated with angiogenesis and poor prognosis in pulmonary adenocarcinoma. Cancer. (2000) 88:2686–92. doi: 10.1002/1097-0142(20000615)88:12<2686::AID-CNCR6>3.0.CO;2-6, PMID: 10870050

[49] Imada A Shijubo N Kojima H Abe S . Mast cells correlate with angiogenesis and poor outcome in stage I lung adenocarcinoma. Eur Respir J. (2000) 15:1087–93. doi: 10.1034/j.1399-3003.2000.01517.x, PMID: 10885428

[50] Coussens LM Raymond WW Bergers G Laig-Webster M Behrendtsen O Werb Z . Inflammatory mast cells up-regulate angiogenesis during squamous epithelial carcinogenesis. Genes Dev. (1999) 13:1382–97. doi: 10.1101/gad.13.11.1382, PMID: 10364156 PMC316772

[51] Nagata M Shijubo N Walls AF Ichimiya S Abe S Sato N . Chymase-positive mast cells in small sized adenocarcinoma of the lung. Virchows Arch. (2003) 443:565–73. doi: 10.1007/s00428-003-0842-y, PMID: 12827514

[52] Rudolph MI Boza Y Yefi R Luza S Andrews E Penissi A . The influence of mast cell mediators on migration of SW756 cervical carcinoma cells. J Pharmacol Sci. (2008) 106:208–18. doi: 10.1254/jphs.FP0070736, PMID: 18296861

[53] Cianchi F Cortesini C Schiavone N Perna F Magnelli L Fanti E . The role of cyclooxygenase-2 in mediating the effects of histamine on cell proliferation and vascular endothelial growth factor production in colorectal cancer. Clin Cancer Res. (2005) 11:6807–15. doi: 10.1158/1078-0432.CCR-05-0675, PMID: 16203768

[54] Welsh TJ Green RH Richardson D Waller DA O’Byrne KJ Bradding P . Macrophage and mast-cell invasion of tumor cell islets confers a marked survival advantage in non-small-cell lung cancer. J Clin Oncol. (2005) 23:8959–67. doi: 10.1200/JCO.2005.01.4910, PMID: 16219934

[55] Nakae S Suto H Iikura M Kakurai M Sedgwick JD Tsai M . Mast cells enhance T cell activation: importance of mast cell costimulatory molecules and secreted TNF. J Immunol. (2006) 176:2238–48. doi: 10.4049/jimmunol.176.4.2238, PMID: 16455980

[56] Lee JM Lee MH Garon E Goldman JW Salehi-Rad R Baratelli FE . Phase I trial of intratumoral injection of CCL21 gene-Modified dendritic cells in lung cancer elicits tumor-Specific immune responses and CD8(+) T-cell infiltration. Clin Cancer Res. (2017) 23:4556–68. doi: 10.1158/1078-0432.CCR-16-2821, PMID: 28468947 PMC5599263

[57] Henry CJ Ornelles DA Mitchell LM Brzoza-Lewis KL Hiltbold EM . IL-12 produced by dendritic cells augments CD8+ T cell activation through the production of the chemokines CCL1 and CCL17. J Immunol. (2008) 181:8576–84. doi: 10.4049/jimmunol.181.12.8576, PMID: 19050277 PMC2716729

[58] Colonna M Trinchieri G Liu YJ . Plasmacytoid dendritic cells in immunity. Nat Immunol. (2004) 5:1219–26. doi: 10.1038/ni1141, PMID: 15549123

[59] Faget J Bendriss-Vermare N Gobert M Durand I Olive D Biota C . ICOS-ligand expression on plasmacytoid dendritic cells supports breast cancer progression by promoting the accumulation of immunosuppressive CD4+ T cells. Cancer Res. (2012) 72:6130–41. doi: 10.1158/0008-5472.CAN-12-2409, PMID: 23026134

[60] Palamara F Meindl S Holcmann M Lührs P Stingl G Sibilia M . Identification and characterization of pDC-like cells in normal mouse skin and melanomas treated with imiquimod. J Immunol. (2004) 173:3051–61. doi: 10.4049/jimmunol.173.5.3051, PMID: 15322165

[61] Jin S Deng Y Hao JW Li Y Liu B Yu Y . NK cell phenotypic modulation in lung cancer environment. PLoS One. (2014) 9:e109976. doi: 10.1371/journal.pone.0109976, PMID: 25299645 PMC4192363

[62] Wang C Yu Q Song T Wang Z Song L Yang Y . The heterogeneous immune landscape between lung adenocarcinoma and squamous carcinoma revealed by single-cell RNA sequencing. Signal Transduct Target Ther. (2022) 7:289. doi: 10.1038/s41392-022-01130-8, PMID: 36008393 PMC9411197

[63] Li J Li H Zhang C Zhang C Wang H . Integrative analysis of genomic alteration, immune cells infiltration and prognosis of lung squamous cell carcinoma (LUSC) to identify smoking-related biomarkers. Int Immunopharmacol. (2020) 89:107053. doi: 10.1016/j.intimp.2020.107053, PMID: 33045568

[64] Lin M Luo H Liang S Chen J Liu A Niu L . Pembrolizumab plus allogeneic NK cells in advanced non-small cell lung cancer patients. J Clin Invest. (2020) 130:2560–9. doi: 10.1172/JCI132712, PMID: 32027620 PMC7190908

[65] He S Yin T Li D Gao X Wan Y Ma X . Enhanced interaction between natural killer cells and lung cancer cells: involvement in gefitinib-mediated immunoregulation. J Transl Med. (2013) 11:186. doi: 10.1186/1479-5876-11-186, PMID: 23937717 PMC3766712

[66] Rosenberg SA Lotze MT Muul LM Leitman S Chang AE Ettinghausen SE . Observations on the systemic administration of autologous lymphokine-activated killer cells and recombinant interleukin-2 to patients with metastatic cancer. N Engl J Med. (1985) 313:1485–92. doi: 10.1056/NEJM198512053132327, PMID: 3903508

[67] Hermanson DL Kaufman DS . Utilizing chimeric antigen receptors to direct natural killer cell activity. Front Immunol. (2015) 6:195. doi: 10.3389/fimmu.2015.00195, PMID: 25972867 PMC4412125

[68] Asano R Nakayama M Kawaguchi H Kubota T Nakanishi T Umetsu M . Construction and humanization of a functional bispecific EGFR × CD16 diabody using a refolding system. FEBS J. (2012) 279:223–33. doi: 10.1111/j.1742-4658.2011.08417.x, PMID: 22074399

[69] Lulla AR Akli S Karakas C Caruso JA Warma LD Fowlkes NW . Neutrophil elastase remodels mammary tumors to facilitate lung metastasis. Mol Cancer Ther. (2024) 23:492–506. doi: 10.1158/1535-7163.MCT-23-0414, PMID: 37796181 PMC10987287

[70] Fridlender ZG Sun J Kim S Kapoor V Cheng G Ling L . Polarization of tumor-associated neutrophil phenotype by TGF-beta: “N1” versus “N2” TAN. Cancer Cell. (2009) 16:183–94. doi: 10.1016/j.ccr.2009.06.017, PMID: 19732719 PMC2754404

[71] Chung JY Tang PC Chan MK Xue VW Huang XR Ng CS . Smad3 is essential for polarization of tumor-associated neutrophils in non-small cell lung carcinoma. Nat Commun. (2023) 14:1794. doi: 10.1038/s41467-023-37515-8, PMID: 37002229 PMC10066366

[72] Zhang D Wang X Li W Wan D Zhou Y Ma C . A single-cell atlas-inspired hitchhiking therapeutic strategy for acute pancreatitis by restricting ROS in neutrophils. Adv Mater. (2025) 37:e2502200. doi: 10.1002/adma.202502200, PMID: 40395143

[73] Lu F Verleg S Groven RVM Poeze M van Griensven M Blokhuis TJ . Is there a role for N1-N2 neutrophil phenotypes in bone regeneration? A systematic review. Bone. (2024) 181:117021. doi: 10.1016/j.bone.2024.117021, PMID: 38253189

[74] Yang J Xie Y Xia Z Ji S Yang X Yue D . HucMSC-exo induced N2 polarization of neutrophils: implications for angiogenesis and tissue restoration in wound healing. Int J Nanomedicine. (2024) 19:3555–75. doi: 10.2147/IJN.S458295, PMID: 38638364 PMC11024985

[75] Tyagi A Sharma S Wu K Wu SY Xing F Liu Y . Retraction Note: Nicotine promotes breast cancer metastasis by stimulating N2 neutrophils and generating pre-metastatic niche in lung. Nat Commun. (2025) 16:4729. doi: 10.1038/s41467-025-59975-w, PMID: 40404609 PMC12098655

[76] Ma Y Wei J He W Ren J . Neutrophil extracellular traps in cancer. MedComm (2020). (2024) 5:e647. doi: 10.1002/mco2.647, PMID: 39015554 PMC11247337

[77] Najmeh S Cools-Lartigue J Rayes RF Gowing S Vourtzoumis P Bourdeau F . Neutrophil extracellular traps sequester circulating tumor cells via β1-integrin mediated interactions. Int J Cancer. (2017) 140:2321–30. doi: 10.1002/ijc.30635, PMID: 28177522

[78] Wang Y Liu F Chen L Fang C Li S Yuan S . Neutrophil extracellular traps (NETs) promote non-Small cell lung cancer metastasis by suppressing lncRNA MIR503HG to activate the NF-κB/NLRP3 inflammasome pathway. Front Immunol. (2022) 13:867516. doi: 10.3389/fimmu.2022.867516, PMID: 35707534 PMC9190762

[79] Zou JM Qin J Li YC Wang Y Li D Shu Y . IL-35 induces N2 phenotype of neutrophils to promote tumor growth. Oncotarget. (2017) 8:33501–14. doi: 10.18632/oncotarget.16819, PMID: 28432279 PMC5464885

[80] Zhang X Huang X Zhang X Lai L Zhu B Lin P . The miR-941/FOXN4/TGF-β feedback loop induces N2 polarization of neutrophils and enhances tumor progression of lung adenocarcinoma. Front Immunol. (2025) 16:1561081. doi: 10.3389/fimmu.2025.1561081, PMID: 40352924 PMC12061992

[81] Mitchell KG Lee Y Deboever N Negrao MV Tran HT Parra E . Intratumoral neutrophil-to-lymphocyte ratio is mirrored by circulating neutrophil-to-lymphocyte ratio in non-small cell lung cancer. J Immunother Cancer. (2025) 13:e011458. doi: 10.1136/jitc-2025-011458, PMID: 40555561 PMC12198785

[82] Matsumoto K Yamamoto Y Shiroyama T Kuge T Mori M Tamiya M . Risk stratification according to baseline and early change in neutrophil-to-Lymphocyte ratio in advanced non-Small cell lung cancer treated with chemoimmunotherapy: A multicenter real-World study. Target Oncol. (2024) 19:757–67. doi: 10.1007/s11523-024-01084-7, PMID: 38990462 PMC11392963

[83] Longueville E Dewolf M Dalstein V Durlach A Vivien A Nawrocki-Raby B . Comparing neutrophil-to-lymphocyte ratio (NLR), absolute neutrophil count (ANC) and derived NLR as predictive biomarkers in first-line immunotherapy for non-small cell lung cancer: a retrospective study. Transl Lung Cancer Res. (2025) 14:1212–30. doi: 10.21037/tlcr-24-808, PMID: 40386737 PMC12082228

[84] Vitkov L Krunić J Dudek J Bobbili MR Grillari J Hausegger B . Vesicular messages from dental biofilms for neutrophils. Int J Mol Sci. (2024) 25:3314. doi: 10.3390/ijms25063314, PMID: 38542287 PMC10970663

[85] Arciprete F Verachi P Martelli F Valeri M Balliu M Guglielmelli P . Inhibition of CXCR1/2 reduces the emperipolesis between neutrophils and megakaryocytes in the Gata1(low) model of myelofibrosis. Exp Hematol. (2023) 121:30–7. doi: 10.1016/j.exphem.2023.02.003, PMID: 36863479 PMC11780361

[86] Kwak JW Nguyen HQ Camai A Huffman GM Mekvanich S Kenney NN . CXCR1/2 antagonism inhibits neutrophil function and not recruitment in cancer. Oncoimmunology. (2024) 13:2384674. doi: 10.1080/2162402X.2024.2384674, PMID: 39076249 PMC11285219

[87] Kargl J Zhu X Zhang H Yang GHY Friesen TJ Shipley M . Neutrophil content predicts lymphocyte depletion and anti-PD1 treatment failure in NSCLC. JCI Insight. (2019) 4:e130850. doi: 10.1172/jci.insight.130850, PMID: 31852845 PMC6975266

[88] Di Vito C Mikulak J Zaghi E Pesce S Marcenaro E Mavilio D . NK cells to cure cancer. Semin Immunol. (2019) 41:101272. doi: 10.1016/j.smim.2019.03.004, PMID: 31085114

[89] Wu SY Fu T Jiang YZ Shao ZM . Natural killer cells in cancer biology and therapy. Mol Cancer. (2020) 19:120. doi: 10.1186/s12943-020-01238-x, PMID: 32762681 PMC7409673

[90] Ichiki Y Shigematsu Y Baba T Shiota H Fukuyama T Nagata Y . Development of adoptive immunotherapy with KK-LC-1-specific TCR-transduced γδT cells against lung cancer cells. Cancer Sci. (2020) 111:4021–30. doi: 10.1111/cas.14612, PMID: 32780528 PMC7648040

[91] Sarode P Zheng X Giotopoulou GA Weigert A Kuenne C Günther S . Reprogramming of tumor-associated macrophages by targeting β-catenin/FOSL2/ARID5A signaling: A potential treatment of lung cancer. Sci Adv. (2020) 6:eaaz6105. doi: 10.1126/sciadv.aaz6105, PMID: 32548260 PMC7274802

[92] Deligne C Murdamoothoo D Gammage AN Gschwandtner M Erne W Loustau T . Matrix-Targeting immunotherapy controls tumor growth and spread by switching macrophage phenotype. Cancer Immunol Res. (2020) 8:368–82. doi: 10.1158/2326-6066.CIR-19-0276, PMID: 31941671 PMC7611136

[93] Wang QT Nie Y Sun SN Lin T Han RJ Jiang J . Tumor-associated antigen-based personalized dendritic cell vaccine in solid tumor patients. Cancer Immunol Immunother. (2020) 69:1375–87. doi: 10.1007/s00262-020-02496-w, PMID: 32078016 PMC11027674

[94] Ma H Zhang Z Hu Q Chen H Wu G Zhou Y . Shedding light on macrophage immunotherapy in lung cancer. J Cancer Res Clin Oncol. (2023) 149:8143–52. doi: 10.1007/s00432-023-04740-z, PMID: 37052632 PMC11797968

[95] Asadollahi E Zomorodipour A Soheili ZS Jahangiri B Sadeghizadeh M . Development of a multi-neoepitope vaccine targeting non-small cell lung cancer through reverse vaccinology and bioinformatics approaches. Front Immunol. (2025) 16:1521700. doi: 10.3389/fimmu.2025.1521700, PMID: 40453095 PMC12122770

[96] Lotze MT Maeurer M Quezada SA Coukos G . Lung cancer adoptive cell therapy: inspiring TIL ACT comes center stage. Cancer Discov. (2024) 14:1366–8. doi: 10.1158/2159-8290.CD-24-0645, PMID: 39091204

[97] Ingels J De Cock L Stevens D Mayer RL Théry F Sanchez GS . Neoantigen-targeted dendritic cell vaccination in lung cancer patients induces long-lived T cells exhibiting the full differentiation spectrum. Cell Rep Med. (2024) 5:101516. doi: 10.1016/j.xcrm.2024.101516, PMID: 38626769 PMC11148567

[98] Li X Zhu YJ Xue Y Chen TT Sun XK Shi HY . Neoantigen-based immunotherapy in lung cancer: advances, challenges and prospects. Cancers (Basel). (2025) 17:1953. doi: 10.3390/cancers17121953, PMID: 40563603 PMC12190263

[99] Shah P Forget MA Frank ML Jiang P Sakellariou-Thompson D Federico L . Combined IL-2, agonistic CD3 and 4-1BB stimulation preserve clonotype hierarchy in propagated non-small cell lung cancer tumor-infiltrating lymphocytes. J Immunother Cancer. (2022) 10:e003082. doi: 10.1136/jitc-2021-003082, PMID: 35110355 PMC8811607

[100] Abodunrin F Olson DJ Emehinola O Bestvina CM . Adopting tomorrow’s therapies today: a perspective review of adoptive cell therapy in lung cancer. Ther Adv Med Oncol. (2025) 17:17588359251320280. doi: 10.1177/17588359251320280, PMID: 40012708 PMC11863254

[101] Xu M Xue B Wang Y Wang D Gao D Yang S . Temperature-feedback nanoplatform for NIR-II penta-modal imaging-guided synergistic photothermal therapy and CAR-NK immunotherapy of lung cancer. Small. (2021) 17:e2101397. doi: 10.1002/smll.202101397, PMID: 34159726

[102] Gemelli M Noonan DM Carlini V Pelosi G Barberis M Ricotta R . Overcoming resistance to checkpoint inhibitors: natural killer cells in non-small cell lung cancer. Front Oncol. (2022) 12:886440. doi: 10.3389/fonc.2022.886440, PMID: 35712510 PMC9194506

[103] Rodriguez-Garcia A Lynn RC Poussin M Eiva MA Shaw LC O’Connor RS . CAR-T cell-mediated depletion of immunosuppressive tumor-associated macrophages promotes endogenous antitumor immunity and augments adoptive immunotherapy. Nat Commun. (2021) 12:877. doi: 10.1038/s41467-021-20893-2, PMID: 33563975 PMC7873057

[104] Zhang Y Zhang C He M Xing W Hou R Zhang H . Co-expression of IL-21-Enhanced NKG2D CAR-NK cell therapy for lung cancer. BMC Cancer. (2024) 24:119. doi: 10.1186/s12885-023-11806-1, PMID: 38263004 PMC10807083

[105] Hou J Xie S Gao J Jiang T Zhu E Yang X . NK cell transfer overcomes resistance to PD-(L)1 therapy in aged mice. Exp Hematol Oncol. (2024) 13:48. doi: 10.1186/s40164-024-00511-9, PMID: 38725070 PMC11080179

[106] De Rosa C Morgillo F Amato L Iommelli F De Rosa V Tirino V . DNA-PK inhibition sustains the antitumor innate immune response in small cell lung cancer. iScience. (2025) 28:111943. doi: 10.1016/j.isci.2025.111943, PMID: 40034862 PMC11875153

[107] Zhi L Zhang Z Gao Q Shang C He W Wang Y . CAR-NK cells with dual targeting of PD-L1 and MICA/B in lung cancer tumor models. BMC Cancer. (2025) 25:337. doi: 10.1186/s12885-025-13780-2, PMID: 40000974 PMC11853679

[108] Wen SWC Nederby L Andersen RF Hansen TS Nyhus CH Hilberg O . NK cell activity and methylated HOXA9 ctDNA as prognostic biomarkers in patients with non-small cell lung cancer treated with PD-1/PD-L1 inhibitors. Br J Cancer. (2023) 129:135–42. doi: 10.1038/s41416-023-02285-z, PMID: 37137997 PMC10307873

[109] Wisdom AJ Hong CS Lin AJ Xiang Y Cooper DE Zhang J . Neutrophils promote tumor resistance to radiation therapy. Proc Natl Acad Sci U S A. (2019) 116:18584–9. doi: 10.1073/pnas.1901562116, PMID: 31462499 PMC6744874

[110] Gong P Ding Y Li W Yang J Su X Tian R . Neutrophil-Driven M2-Like macrophages are critical for skin fibrosis in a systemic sclerosis model. J Invest Dermatol. (2024) 144:2426–39.e2423. doi: 10.1016/j.jid.2024.03.031, PMID: 38580106

[111] Song C Li H Li Y Dai M Zhang L Liu S . NETs promote ALI/ARDS inflammation by regulating alveolar macrophage polarization. Exp Cell Res. (2019) 382:111486. doi: 10.1016/j.yexcr.2019.06.031, PMID: 31255598

[112] Hu G Wang S Cheng P . Tumor-infiltrating tryptase(+) mast cells predict unfavorable clinical outcome in solid tumors. Int J Cancer. (2018) 142:813–21. doi: 10.1002/ijc.31099, PMID: 29023696

[113] Passaro A Brahmer J Antonia S Mok T Peters S . Managing resistance to immune checkpoint inhibitors in lung cancer: treatment and novel strategies. J Clin Oncol. (2022) 40:598–610. doi: 10.1200/JCO.21.01845, PMID: 34985992

[114] Attili I Del Re M Guerini-Rocco E Crucitta S Pisapia P Pepe F . The role of molecular heterogeneity targeting resistance mechanisms to lung cancer therapies. Expert Rev Mol Diagn. (2021) 21:757–66. doi: 10.1080/14737159.2021.1943365, PMID: 34278933

[115] Exposito F Redrado M Houry M Hastings K Molero-Abraham M Lozano T . PTEN loss confers resistance to anti-PD-1 therapy in non-small cell lung cancer by increasing tumor infiltration of regulatory T cells. Cancer Res. (2023) 83:2513–26. doi: 10.1158/0008-5472.CAN-22-3023, PMID: 37311042

[116] Zhang D Zhan D Zhang R Sun Y Duan C Yang J . Treg-derived TGF-β1 dampens cGAS-STING signaling to downregulate the expression of class I MHC complex in multiple myeloma. Sci Rep. (2024) 14:11593. doi: 10.1038/s41598-024-62298-3, PMID: 38773213 PMC11109281

[117] Simpson KL Rothwell DG Blackhall F Dive C . Challenges of small cell lung cancer heterogeneity and phenotypic plasticity. Nat Rev Cancer. (2025) 25:447–62. doi: 10.1038/s41568-025-00803-0, PMID: 40211072

[118] Wu F Fan J He Y Xiong A Yu J Li Y . Single-cell profiling of tumor heterogeneity and the microenvironment in advanced non-small cell lung cancer. Nat Commun. (2021) 12:2540. doi: 10.1038/s41467-021-22801-0, PMID: 33953163 PMC8100173

[119] Suresh K Voong KR Shankar B Forde PM Ettinger DS Marrone KA . Pneumonitis in non-Small cell lung cancer patients receiving immune checkpoint immunotherapy: incidence and risk factors. J Thorac Oncol. (2018) 13:1930–9. doi: 10.1016/j.jtho.2018.08.2035, PMID: 30267842

[120] Sacchi de Camargo Correia G Pai T Li S Connor D Zhao Y Lou Y . Immune-related adverse events in patients with lung cancer. Curr Oncol Rep. (2023) 25:1259–75. doi: 10.1007/s11912-023-01462-w, PMID: 37782426

[121] Chen X Gao A Zhang F Yang Z Wang S Fang Y . ILT4 inhibition prevents TAM- and dysfunctional T cell-mediated immunosuppression and enhances the efficacy of anti-PD-L1 therapy in NSCLC with EGFR activation. Theranostics. (2021) 11:3392–416. doi: 10.7150/thno.52435, PMID: 33537094 PMC7847666

